# Toward a Knowledge-Based System for African Traditional Herbal Medicine: A Design Science Research Approach

**DOI:** 10.3389/frai.2022.856705

**Published:** 2022-03-09

**Authors:** Samuel Nii Odoi Devine, Emmanuel Awuni Kolog, Roger Atinga

**Affiliations:** ^1^Department of Information and Communication Technology, Presbyterian University College, Abetifi, Ghana; ^2^Department of Operations and Management Information Systems, University of Ghana, Accra, Ghana; ^3^Department of Public Administration and Health Services Management, University of Ghana, Accra, Ghana

**Keywords:** knowledge base, ontology, traditional herbal medicine, design science research, machine learning

## Abstract

This article illustrates a design approach for capturing, storing, indexing, and search of African traditional herbal medicine (ATHMed) framed on a hybrid-based knowledge model for efficient preservation and retrieval. By the hybrid approach, the framework was developed to include both the use of machine learning and ontology-based techniques. The search pattern considers ontology design and machine learning techniques for extracting ATHMed data. The framework operates on a semantically annotated corpus and delivers a contextual and multi-word search pattern against its knowledge base. In line with design science research, preliminary data were collected in this study, and a proposed strategy was developed toward processing, storing and retrieving data. While reviewing literature and interview data to reflect on the existing challenges, these findings suggest the need for a system with the capability of retrieving and archiving ATHMed in Ghana. This study contributes to SDG 3 by providing a model and conceptualizing the implementation of ATHMed. We, therefore, envision that the framework will be adopted by relevant stakeholders for the implementation of efficient systems for archival and retrieval of ATHMed.

## Introduction

Countries worldwide are striving to promote good health and wellbeing for their citizens. Achieving universal healthcare by meeting United Nations' (UN) Sustainable Development Goal (SDG) 3 is the focus of many countries. The SDGs provide an ambitious and comprehensive plan of action for ending the injustices that underpin poor health and development outcomes (SDGF, 2020). It also seek to achieve universal health coverage and provide access to safe and effective medicines and vaccines for all (SDGF, 2020). The African continent is undoubtedly one of the poorest continents with poor health conditions, though it can boost of rich natural resources. It is therefore imperative for the continent to harness its own potential and available resources, including herbal medicine, to realize good health outcomes instead of relying on the western countries for solutions. This study contributes to the realization of the SDG 3 by developing a computational framework for the effective and efficient implementation of ATHMed.

In the spirit of sustainability and standardization, WHO (2019) has thrown the challenge to the global community to build knowledge bases for the management of traditional herbal medicine in its Traditional Medicine Strategy (WHA62.13) 2014–2023 (WHO, 2019). Historically, several approaches have been adopted to assist in properly and sustainably securing, in addition, to efficiently sharing knowledge. Current practices and the nature of the challenges presented point toward employing knowledge management approaches which largely is dominated by computer-based solutions. Therefore, a computing-focused solution is advocated to be most suited to answer this call by adopting sustainable, progressive, and novel approaches to help safeguard ATHMed knowledge (Devine et al., 2019). The value of computer-based approaches extends through history as the impact leads to sustainability. This article discusses a hybrid approach involving the use of machine learning and ontology approaches for preservation and retrieval of THMed knowledge. It further discusses preliminary work on data collection and how it influences the design of the proposed ATHMed KB framework. Additionally, this article illustrates search approaches influenced by natural language processing techniques that can be adopted toward the preservation and retrieval of ATHMed knowledge acquired from “deep smart” knowledge bearers using computing strategies. This is aimed at presenting explicit, well-articulated ATHMed knowledge for formal training and research in Medical Institutions of Higher Learning while promoting use in the herbal business industry. Adopting a design science research allows a preliminary understanding of the herbal medicine ecosystem. Therefore, data was elicited from stakeholders of ATHMed.

For many centuries, diverse methods have been applied in different cultures to provide healthcare. Popular amongst these forms of practice in Ghana is the orthodox and traditional herbal treatments. Traditional herbal medicine can help many countries to achieve SDG3, especially in the developing context. Traditional herbal medicine was the first method of healthcare practice in Ghana (Appiah et al., 2019). Traditional herbal medicine (THMed) with other complementary and alternative healthcare practices are widely prevalent in many regions in Africa (James et al., 2018; WHO, 2019). The 2019 World Health Organization (WHO) report on traditional and complementary medicine indicates a growing interest in the area, which is undergoing revival, “given the unique health challenges of the twenty-first century” (WHO, 2019, p. 5). Interestingly, the WHO report also shows many countries have formulated national policies and regulation, and developed programmes covering herbal medicine, thus interest in this healthcare alternative is growing. This prominence has encouraged WHO and other multilateral organizations to play “key roles in supporting capacity development in the traditional medicine sector, including the development of local manufacturing” (WHO, 2021, para. 9), leading to WHO, the African Union Commission and Africa CDC jointly launching the Regional Expert Advisory Committee on Traditional Medicine for COVID-19 Response (WHO, 2021). It is also reported that many of these herbal users are in developing countries due to the perceived potency, ease of use and low cost (James et al., 2018; Appiah et al., 2019; WHO, 2019). In Ghana, though orthodox medicine is highly patronized, it is estimated that 70% (Amoah et al., 2014) to 80–99% (WHO, 2019, p. 71) of its population are claimed to use herbal medicine to treat their ailments and complement their healthcare needs.

Reportedly, yet likely, the knowledge is habitually presented in part which in effect leads to half-baked herbal practitioners providing questionable medical care, implying medication prepared is likely not to perform what it promises, as such dangerous to the health of ATHMed patrons (Yeboah, 2000). Amoah et al. (2014) indicate that herbal practitioners often poorly communicate and hardly document their knowledge resulting in knowledge failing to be developed or maturing into an accepted treatment, practice or medication, or that knowledge is eventually lost. This approach to knowledge transfer is deemed to be tacit, as it consists of the ideas, experiences and skill set possessed by a person which is often difficult to access and transfer (Chugh, 2018, p. 2; Nonaka and Takeuchi, 1995). This tacit approach is inefficient and not appropriate for long-term preservation and use of knowledge. This probably expounds the reason for ATHMed knowledge being lost with time. There are reported instances of misinformation, misappropriation and abuse of herbal knowledge especially associated with the traditional health knowledge (Yeboah, 2000, p. 208). This raises a cause for concern due to poor ATHMed documentation. The lack of appropriate documentation for ATHMed practice involving the selection of ingredients, preparation methods, and their administration has brought the quality, efficacy, and safety of ATHMed into suspicion (Chikezie and Ojiako, 2015). Regrettably, this has formed doubt in the patronage of herbal products and medication. The relevance of knowledge preservation cannot be downplayed as it holds strategic influence on the growth, sustenance, and competitive advantage of any environment, organization, or people for that matter (Davenport and Prusak, 1998; Xue, 2017) Consequently, steps to mitigate and possibly correct the challenge bedeviling the practice of proper documentation associated with ATHMed appropriate strategies are necessary to salvage the situation. To this end, there have been many calls seeking to improve and formalize the practice of proper preparation and preservation of such practices (WHO, 2019).

## Literature Review

### Traditional Herbal Medicine in Ghana

Traditional medicine, according to WHO, involves “knowledge, skill, and practices based on the theories, beliefs, and experiences indigenous to different cultures, whether explicable or not, used in the maintenance of health as well as in the prevention, diagnosis, improvement or treatment of physical and mental illness” (WHO, 2019, 2020, p. 44). In the case of herbal medicine, it involves “herbs, herbal materials, herbal preparations and finished herbal products that contain as active ingredients parts of plants, or other plant materials, or combinations” (WHO, 2019, p. 29). Tilburt and Kaptchuk (2008) situate traditional herbal medicines to imply “naturally occurring, plant-derived substances with minimal or no industrial processing that have been used to treat illness within local or regional healing practices”.

A larger proportion of herbal medicine patrons are in the rural areas (Amoah et al., 2014; James et al., 2018; Appiah et al., 2019). Research into the domain of traditional and herbal medicine has gained renewed interest (Appiah et al., 2019; WHO, 2019). However, despite its relevance, the knowledge regarding ATHMed dissemination is verbal, remains largely undocumented and poorly recorded (Chikezie and Ojiako, 2015; Boadu and Asase, 2017; Appiah et al., 2019). Furthermore, the processes of identifying, preparing and administering herbal medicine have come under critique. Cases of misapplication of medication, perceived adverse side effects, safety, efficacy, and quality, with mistrust and dosage standardization in the use of ATHMed have been cited in reports and literature (Yeboah, 2000; Chikezie and Ojiako, 2015; Boateng et al., 2016; James et al., 2018; Appiah et al., 2019). A major factor contributing to this is the poor documentation, or lack thereof, often associated with the practice of African traditional herbal medicine (Appiah et al., 2019). The knowledge bearers of herbal practices, thus the herbal medicine practitioners, often have an exclusive hold on their knowledge. In the act to protect their trade and knowledge, many ATHMed practitioners, especially in Ghana, prefer to pass on their knowledge to only their close relatives and through generations (Boadu and Asase, 2017; Appiah et al., 2019). Adekannbi et al. (2014) observe that although certain herbal practitioners are willing to part with their knowledge, they intentionally and articulately attempt to transfer their knowledge to assistants who are their close relations. This transfer is often and primarily done through observation and direct practice with little or no documentation (Boateng et al., 2016). Leisurely, the knowledge is presented orally to these ATHMed trainees, who learn by doing and are only exposed to the knowledge at the pace and wish of their trainers who are the sole bearers of the herbal knowledge (Yeboah, 2000; Adekannbi et al., 2014; Soelberg et al., 2015; Boateng et al., 2016; Maluleka and Ngulube, 2018; Appiah et al., 2019). Appiah et al. (2019) emphasize that in-depth knowledge on ATHMed is at the brink of extinction due to the predominant reliance of oral mode of transmission. Sadly, this often leads to the likelihood of such knowledge being either lost or failing to be developed over time (Amoah et al., 2014). In the case where the knowledge is not shared at all, the practitioner eventually dies with the knowledge (Appiah et al., 2019).

As widely observed in literature, there exists a number of patrons for traditional herbal medicine in Ghana (Yeboah, 2000; Boadu and Asase, 2017). The use of herbal medicine and its means of preparation have gained the attention of many stakeholders including governmental institutions, health institutions, and academia in addition to its practitioners and patrons. This is due to the patronage and role it plays in providing and supplementing healthcare needs of Ghanaians who acquire such THMed from licensed shops that sell herbal products and herbal clinics (WHO, 2019: p70). However, as earlier indicated, the practice and its products have also come into question placing doubt on ATHMed products which can also be associated with standardized practices of drug preparation and administration (Boateng et al., 2016; Boadu and Asase, 2017). This has influenced diverse approaches to protect not only the citizenry but also assist in producing practitioners who are well-trained in the area. This is effectively possible *via* the availability of knowledge that is well-defined or explicitly documented. The Government of Ghana realizing such a need has set up the Centre for Scientific Research into Plant Medicine to lead the way in the preparation and standardization of herbal medicine in Ghana (Amoah et al., 2014; Appiah et al., 2019).

In recent times, attempts have been made by Universities in Ghana to train pharmacists in the area of indigenous African herbal medicine treatment. The Kwame Nkrumah University of Science and Technology offers a degree programme in herbal medicine at the undergraduate level. Such training is in an attempt to assist in formalizing the training of professionals in herbal medicine. However, their focus has mainly been to help curb the challenge related to the Ghanaian traditional herbal medicine practitioner's accurate measurement of ingredients for drug preparation with issues on the quality. The universities also strive to provide strategies for long-term preservation, appropriate forms of administration, and administering the right dosage of such herbal drugs. For instance, the University of Ghana in 2016 organized a 2-day face-to-face training programme for manufacturers of herbal products/food supplements, focusing on the improvement of safety and efficacy, evaluation of raw materials, toxicological assessment, quality, and standardization in Ghana. These interventions seek to harness the potential of herbal medicine, providing orthodox and scientific approaches to standardizing and safeguarding the knowledge associated with it and the practices in applying medication. This attempt also assists in documenting and preserving practices and medications associated with African traditional herbal medicines which hitherto was in the domain of the practitioner. Failure to undertake such interventions would, in the end, lead to loss of these indigenous yet efficient medicinal approaches (Boadu and Asase, 2017). It is for this call that we proposed this framework, targeted at providing a knowledge-based system for the efficient, sustainable approach to safeguarding ATHMed knowledge to a more explicit form.

### Knowledge Management Approaches

Throughout history, attempts and varying approaches have been developed and implemented to safeguard, as well as disseminate knowledge. This is to ensure posterity and present benefit from works, acts, processes, information and all relevant data that will help improve or maintain personal to organizational and societal development needs. These views are critical and relevant toward the debate on the predominantly inefficient ATHMed knowledge preservation practices as revealed in literature (Yeboah, 2000; Adekannbi et al., 2014; Maluleka and Ngulube, 2018). Knowledge, as an asset in whichever form, is regarded as key to any organization's present and future growth, and competitive advantage especially in the 21st century (Xue, 2017). Different views have been suggested (Bolisani and Bratianu, 2018) on what “knowledge” is, though many of these definitions still point toward Plato's postulation of knowledge as being “justified true belief” (Nonaka and Takeuchi, 1995). Knowledge, however, can be classically expressed in contemporary times as encompassing a flexible fusion of expert opinion, context-based information, personal exposure, practices and thoughts or reasoned actions, values and norms, and procedures. It is often viewed as inherent and defined by the people who own or use it (knowledge workers) (Davenport and Prusak, 1998), as in the case of many THMed practitioners. This suggests that knowledge can be explicit or tacit in nature (Nonaka and Von Krogh, 2009). By implication, the context and timing, depth and/or coverage and volume may contribute to its value and relevance to any individual or organization's actions and decision-making processes (Meacham, 1983; Kakabadse and Kakabadse, 2001). In this light, it is imperative that, knowledge, in whichever form (explicit or tacit), must be carefully managed, safeguarded and disseminated, thus yielding the relevance of knowledge management.

Knowledge Management (KM) has evolved since its inception as a concept in the 1990s into a full academic discipline, taking different dimensions of classification and study (Xu et al., 2008; Girard and Girard, 2015). Advancement in technology, with the coming of age of computing, has greatly impacted on KM practices. According to Girard and Girard (2015) the “classic and most cited” epistemological attempts in defining KM could be attributed to Davenport and Prusak (1998), and O'Dell and Grayson (1998). In attempting to define KM, many academicians and practitioners, from varying points of view, often leaned toward their disciplines and domains of practice or research. From their observations, Girard and Girard (2015) suggest a definition for KM based on 100 sampled definitions spanning various disciplines from extant literature, having regular occurring words including “use, create, share, knowledge, process, organization, and information”. This research, from the foundational definitions, views knowledge management to be an approach, discipline or mechanism encompassing activities or processes that involve the efficient, systematic, meticulous acquisition of knowledge, accessing, evaluating, dissemination, maintenance and management of knowledge, be it tacit or explicit to the benefit of individuals, groups or organizations (O'Dell and Grayson, 1998; Antoniou et al., 2012; Girard and Girard, 2015).

Research has been intensified in the area with new disciplines interacting with the KM concept to provide an encompassing approach to harness and efficiently manage and disseminate such crucial knowledge, especially for mission-critical tasks or projects within organizations (Davenport and Prusak, 1998). Advancement in technology, with the coming of age of computing, has greatly impacted on KM practices, especially in preservation and retrieval. Consequently, various techniques, strategies and concepts in areas such as Artificial intelligence, machine learning, analytics, data mining, amongst others, have been adopted and incorporated in KM and KM Systems (Chen et al., 2005). The approach of preserving knowledge through KB with ontologies has helped reshape the focus of the domain, especially with this ubiquitously rapid growth of knowledge existing on the web and in organizations (El Morr and Subercaze, 2010). KM also has extended from focusing solely on organizational knowledge to personal knowledge of individuals (Wright, 2005) within the organization, seeking to maximize their productivity (Cheong and Tsui, 2010), consciously or unconsciously. As such, a look into prevailing computing approaches employed currently in KM is necessary and the extent to which it applies to THMed.

Mazour (2006, p. 2) expounds knowledge preservation as “a process for maintaining knowledge important to an organization's mission that stores knowledge/information over time and provides the possibility of recall for the future”. This suggests the need for consistent and meticulous efforts in harnessing and documenting such critical knowledge assets. This can be achieved using information technology, thus computer-based solutions, as these are more sustainable and effective toward formalizing and explicit preservation of knowledge than verbal, oral and face-to-face approaches (Panahi et al., 2012; Ashkenas, 2013). Efficient retrieval and dissemination of knowledge, especially tacit knowledge, is heavily influenced by proficient knowledge preservation, which is vital and inevitable (Sarkiunaite and Kriksciuniene, 2005). KP processes encompass three stages involving the ability to select, store, and actualize (Probst et al., 2006) knowledge. Information technology tools identified to facilitate the KP processes are commonly associated with capturing and sharing systems (Davidavičiene and Raudeliuniene, 2010), in consequence, are focused on preservation and retrieval which involves propagation. As a result, this article focuses on the preservation and retrieval of knowledge relating to codification, representation and storage of knowledge (Antonova et al., 2006) based on prior studies to advance the need and strategy for an ATHMed KB implementation.

Extant literature in KB development indicates focus toward intuitive, robust, platform-independent, metadata dependent, semantically driven, contextual domain-based knowledge modeling, reasoning, preservation, and extraction systems. Evidenced from literature, the adoption of machine learning and ontology-based techniques with their supporting technologies were found to be recurring in diverse, multidisciplinary fields including THMed for KB and thus KB system development (Li et al., 2003; Tomai and Spanaki, 2005; Lin et al., 2013; Sanya and Shehab, 2014; Song et al., 2016; Shang et al., 2017). In their study, Song et al. (2016) explored a 3-tier system architectural framework for KB Systems management of manufacturing process knowledge. The researcher proposed an effective reusable management approach to implementing their KB system, *via* a systematic methodology for constructing the KB, indicating the key role of ontology development through an iterative process. In a related study, Shang et al. (2017), by employing a vulnerability-centric ontology-based KB framework strategy, cyber security knowledge existing in some independent KBs and the internet, in text form, can be efficiently integrated, enabling the extraction of cyber security knowledge with the use of both rule-based and machine learning information extraction techniques. This indicates that current research into KB development should be seen pointing toward the inclusion of machine learning and ontology-based technologies.

#### Machine Learning Approaches

Machine Learning (ML) is one of the fastest growing fields in computer science and has been applied diversely (Shalev-Shwartz and Ben-David, 2014). Since the term Machine learning was coined by Arthur L. Samuel, a number of definitions have been given. Shalev-Shwartz and Ben-David (2014) define machine learning as “automated detection of meaningful patterns in data”. Machine learning involves the use of statistical-computational methods to enable a machine (thus computing system) to learn, focused on improving their performance based on experience obtained from some tasks tackled without being explicitly programmed to do so (Mitchell, 2006). Machine learning application and research in both academia and industry, as seen with evidence-based decision-making in areas including healthcare, education, business, etc. (Jordan and Mitchell, 2015), has generated immense interdisciplinary interest in the field.

Machine learning applications involve supervised and unsupervised learning, implemented through classification and clustering, respectively. This article and the proposed ATHMed framework focus on the supervised learning approach. In clustering, the classifier looks for patterns in data. Classification, as a supervised learning technique, centers on the task of learning a function that maps an input (*x*) to an output (*Y*) based on input-output pairs (*x*_*i*_*, y*_*i*_). Classification makes use of the initial data collected to train a machine learning algorithm (classifier) to predict unseen data. Classification involves training and prediction. At the training stage, the labeled data is classified into two forms being, training data, and testing data. With machine learning classification, the initial stages require labeling/ tagging of sample (training) data, thus n-dimensional vectors that have class association labeling. The sample or input data can be labeled *manually* (by a human expert of a specific domain), *automatically* (using software with natural language capabilities) and *semi-automatically* (mixture of an expert and software) (Kolog et al., 2018).

During training, a model for the classification process is generated. To achieve this, it is ensured that large amounts of the sample (training) data is used as input for learning to the algorithm. The training data is typically textual, and thus classification can be done at different levels, being at the word, sentence, and document levels (Kolog et al., 2019). Firstly, feature extraction of instances is performed on the training data using some technique like part-of-speech-tagging to ensure the highest quality of outcome (output) is obtained to feed the classifier. In the process, *stop words* like “*a*”, “*are*”, and “*the*” are removed or minimized to generate a feature. For instance, the statement “*the leaves of the neem tree are used for the treatment of fever*” will become “*leaves neem tree treatment fever*”. Then lemmatization is performed to obtain the base forms of the possible lexemes in the feature leading to the “*leaf neem tree treat fever*”. The resulting outcome is the model.

At the test stage, the machine learning model generated will be given data to test if it is able to appropriately deduce correctly which class the extracted feature belongs to, in essence for it to predict the right output of the data. If this is correctly achieved at a satisfactory stage, learning ceases. Mathematically, for a given input (*x*) and output (*Y*) variables, the input variable is mapped to the output variable, i.e., *Y* = *f*(*x*). The rationale is to as accurate as possible map *via* the machine learning algorithm (model) such that for any new input data (*x*) the model can efficiently predict (≥70% accuracy) the output variables (*Y*) for that data such that the error on the output predicted is minimal. The predictive ability of the classifier (algorithm), thus its quality, can be measured based on its recall, precision or f-measure. Other popular methods for measuring a classifier's quality include Cohen's Kappa scores, Receiver Operating Characteristic (ROC), among others. Several studies indicate the active adoption and integration of machine learning applications in traditional herbal medicine.

#### Ontology-Based Approach

Ontology is considered to have its origin from philosophy. The concept has been applied in different fields of study including computer Science (CS), specifically in the area of Artificial Intelligence (AI) concentrated in the areas of knowledge management, knowledge engineering, information retrieval, natural language processing, intelligent information integration, etc. (Studer et al., 1998; Guarino et al., 2009). In an attempt to define ontology in the context of computer science, varying definition are presented with Studer et al. (1998) postulation being widely accepted. Studer et al. (1998), based on the initial definitions of Gruber (1993) and Borst (1997), define computational ontology as “a formal, explicit specification of a shared conceptualization”. They further elucidated that “a ‘*conceptualisation'* refers to an abstract model of some phenomenon in the world by having identified the relevant concepts of that phenomenon. ‘*Explicit'* means that the type of concepts used, and the constraints on their use are explicitly defined. ‘*Formal'* refers to the fact that the ontology should be machine readable, which excludes natural language. ‘*Shared'* reflects the notion that an ontology captures consensual knowledge, that is, it is not private to some individual, but accepted by a group” (Studer et al., 1998, p. 25). These views fit well with the approach and focus pursued in the proposed ATHMed framework which is approached adopting the hybrid approach (ML and ontology technologies).

Computational ontologies have been identified to be suited for contextual design and representation of dynamic reasoning knowledge (Studer et al., 1998) that requires implementation of semantic components of knowledge management. Computational ontologies enable the formal modeling of a system's structure (Guarino et al., 2009). Thus, in describing things in terms of their allotted category/class and relation within the AI domain, a pragmatic view of an ontology is the ability to represent that which exists or perceived to exist (Guarino et al., 2009). This implies that an ontology can be built or constructed about anything (entity) tangible or intangible so far as their attributes (relation, instances, axioms, etc.) can be defined. Therefore, in considering an ontological approach we seek to produce a domain model that embodies classes/categories, relations, constraints, and instances applicable to that domain. Thus, the possibility of ontologies in the construction of a KBS (Studer et al., 1998), given its application in domain knowledge representation and is also applicable in natural language processing to facilitate automatic extraction of knowledge from text.

There are different types of ontologies including domain, generic, application, representational, method, and task ontologies (Studer et al., 1998). As concerned with this study, domain ontology is deemed suitable for ATHMed since it is targeted at knowledge specific to a given domain. Again, it is observed that as knowledge within an organization (applicable to THMed) evolves into diverse knowledge elements (Studer et al., 1998), novel methods for capturing, storing, retrieving, and sharing such knowledge (especially tacit) is needed, it necessitates the adoption of ontology-based approaches. Ontology technologies have been adopted in recent times in supporting semantic representation of knowledge in software applications and web environment using authoring and rendering tools. It is on this premise, among others in extant literature, that the ontology approach is deemed suitable and relevant for the engineering, capturing, storage, and querying of THMed Knowledge. Several studies have applied machine learning or ontology in knowledge representation and extraction in diverse domains. Arji et al. (2019) identified studies that involved the use of machine learning in traditional medicine. Current literature indicates the use of ontology in KB design and has been applied to the traditional and herbal medicine domain, thus THMed, with few in the African context.

## Methodology

**Figure 1:**
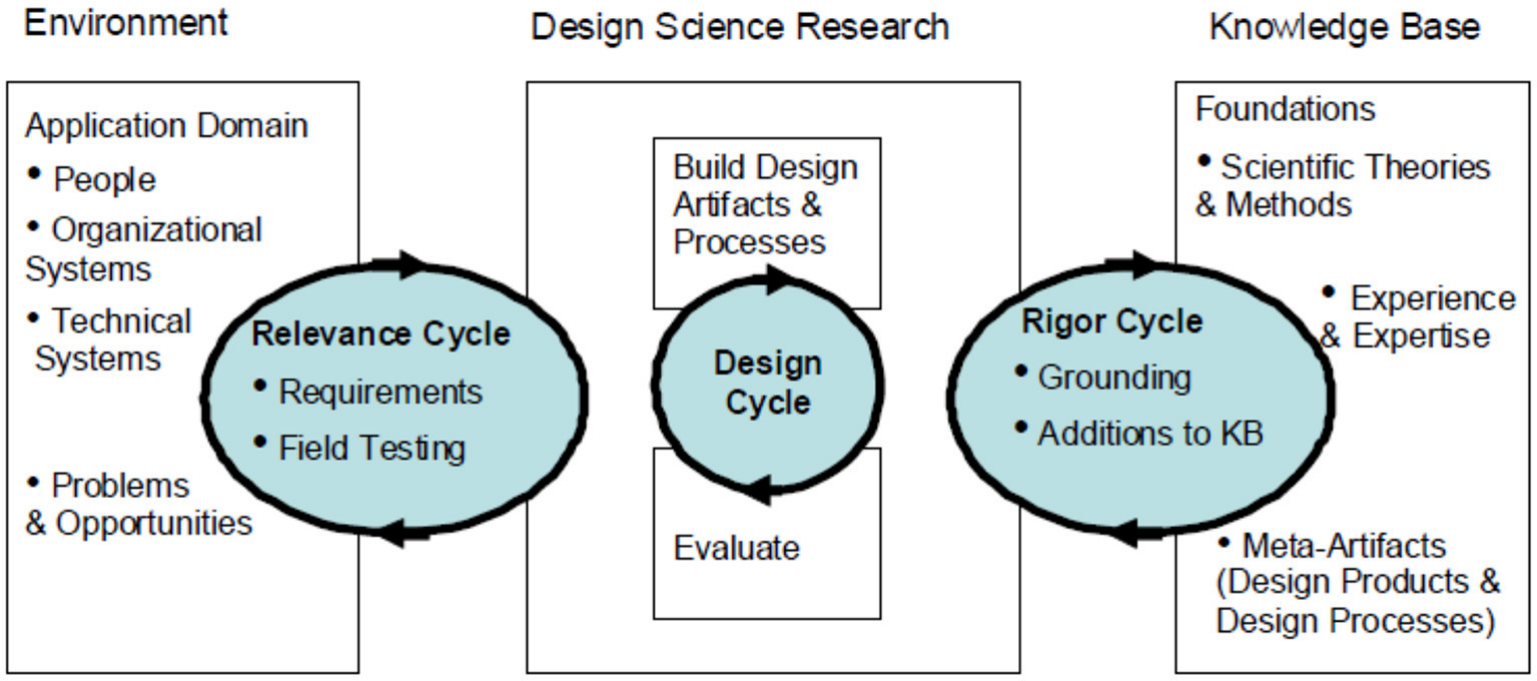
Design science research process (Adopted from Hevner, 2007).

This study adopts a design science research (DSR) process for developing a framework for the ATHMed. Design science research has largely been adopted in several disciplines, especially in the Information System domain. Horváth (2007) and Baskerville et al. (2015) provided two key mandates of DSR: (1) to utilize the gained knowledge to solve problems, create change or improve existing solutions; and (2) to generate new knowledge, insights and theoretical explanations. These mandates go to suggest that DSR strives to understand the real-world problem and find a solution to that problem (Cleven et al., 2009). The real-world problem is simulated, and solution provided in the form of artifact. Artifact development in DSR can be constructs, methods, models and instantiations. DSR recommends the inclusion of the end-users throughout the co-creation of an artifact (constructs, methods, models, and instantiations) (Hevner et al., 2004). The development of DSR artifact undergoes a design process where Hevner et al. (2004), one of the frontiers of DSR, advocated for a co-creation of an artifact with stakeholders. Figure 1 shows a DSR framework developed by Hevner (2007). The framework, as widely been used for artifact development, is made up of three stages: *Environment, Design science*, and *knowledge base*. These stages are not independent but linked iteratively in cycles. These cycles, as indicated Figure 1, are the *relevance, design*, and *rigor* cycles.

The environment constitutes the understanding of the application domain, which include the people, organization and the technical systems. The goal is to formulate objectives for developing solution through design and evaluate process. The design stage involves the actual development of the artifact. However, the design stage undergoes a vertical stepwise process (Design cycle: build and evaluate). The environment and design stages iterate until a desirable outcome is attained and this constitute the relevance cycle. The artifact is expected to add knowledge to the domain for which the study was undertaken. Thus, contributing to the knowledge base largely relies on the developed artifact. The iterative process, at this stage is the rigor cycle.

This article forms part of the relevance and rigor cycles where data was collected to understand the need for the system and the framework for future implementation. The development of the framework relied heavily on the need for it (stakeholders' acceptance). In view of this, the preliminary part of exploring the environment was through data collection. Diverse stakeholders in the herbal medicinal space in Ghana were purposively selected for an interview. The selection was based on their desire to advance research and promote ATHMed. Participants were selected from the Academic institutions advancing herbal medicine, Research institutions (CSIR), Local herbal practitioners, Herbal clinics, Food and Drugs Authority (FDA) and a selection of herbal medicine users in Ghana. The study complied with ethical standards. Thus, Ethical clearance from the University of Ghana ethics board was obtained before undertaking this study. An informed consent agreement form was signed by each participant before the interviews were conducted. A total of 20 stakeholders were interviewed. The interview was semi-structured that allows the participants to express their views by responding to the questions.

## ATHMed Knowledge-Based Framework

This study adopts Initial data collected suggests a keen interest in the development of the computational framework for proper archiving and retrieving of ATHMed. The participants recognized computational systems for ATHMed as a way of contributing to practical knowledge sharing, socio-cultural sustainability concerns and healthcare delivery and accessibility issues especially to patrons and researchers in the domain. On the question of how herbal medicine practitioners are regulated in Ghana, one of the participants blamed the government for negligence in the influx of fake herbal medicine and practitioners in Ghana. One of the participants said:

*“I think the government is not doing enough to check the herbs and the herbal clinics in the country, every day and day out people bring new herbs and just go to the street to sell to the poor Ghanaian. Our country is been poisoned by these acts.”*

Nevertheless, the participants expressed their concerns about the rate at which herbal clinics and herbal practitioners are springing up without proper checks and documentation. We asked a question on their perception regarding a national database for archiving and retrieving traditional herbal medicine. Though the participants did not put the entire blame on the FDA, they rather believe that computational means to help curb the menace of fake herbal medicine is the way to go. One of the participants expressed that:

*“Taken the step to use a more sophisticated technique to help preserve the knowledge in the herbal medicine is the way to go. In fact, I don't entirely blame the authorities who mandated to check fake herbs in the market but making people aware through whatever means will automatically reduce the menace of fake herbs in the system”*

Inthe interview, asked whether there is existing database in pace for archiving herbal medicine. We found out that computational systems are available in specific places, but they are not integrated. These systems are built based on the traditional database system with simple query techniques. One of the participants agreed on the relevance of the development of knowledge-based system.

*“This will be a game changer. I think this will make a work flexible if a knowledge system can be developed to achieve and even help predict what herb to use for a particular ailment”*

On the question of whether the participants will be willing to participate, in terms of building the knowledge-based system, the responses were positive. However, they cautioned that some of the herbs are fake and do not treat the ailment that it has been purported to treat. They, however, recommended for thorough validation with the FDA, an authority mandated to validate drugs before it is put to the market. In future, the various stakeholders will be consulted for practically implementing the framework.

**Figure 2:**
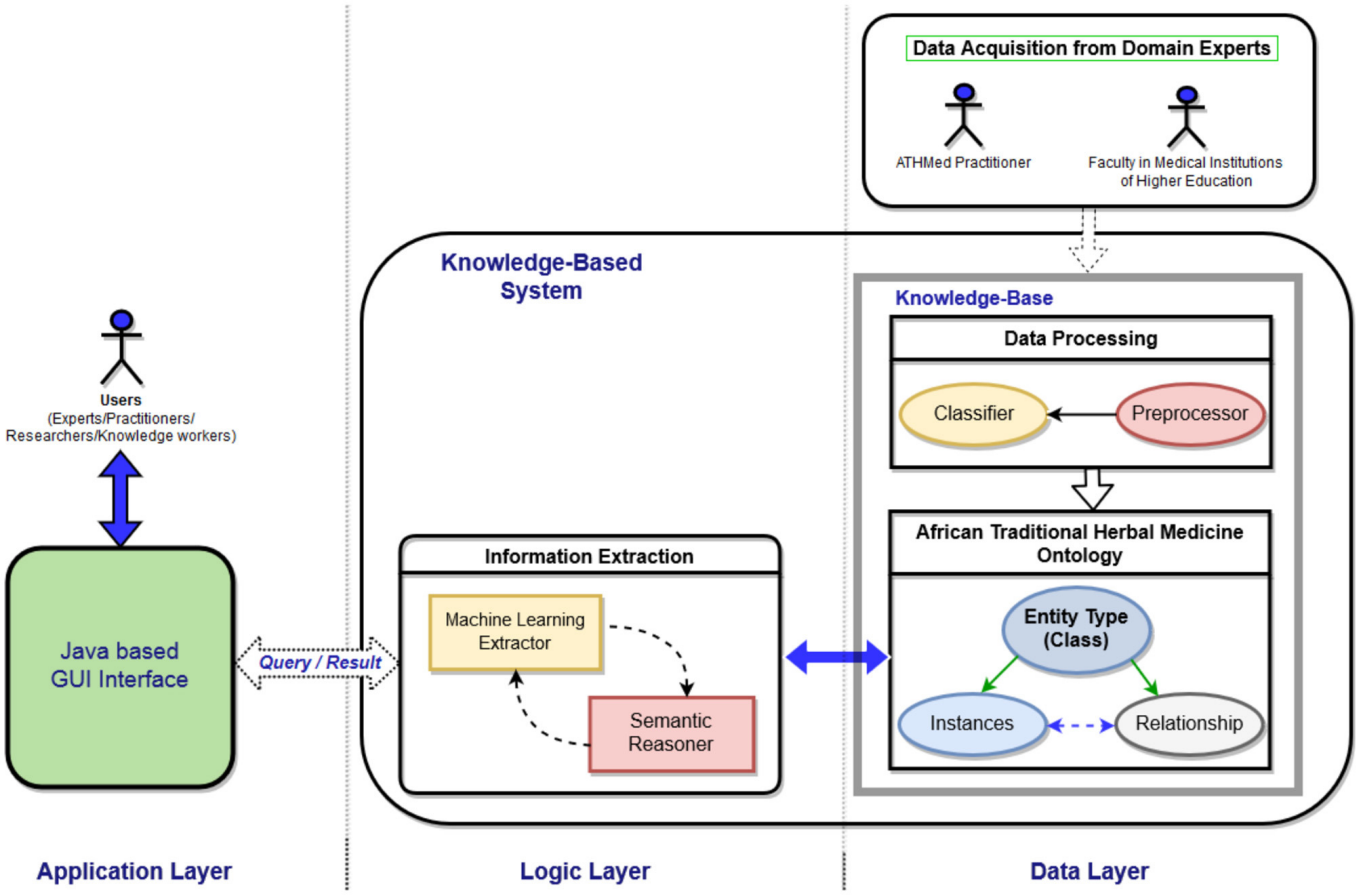
Proposed Knowledge-based Design framework for African traditional herbal medicine (Devine et al., 2019).

The ATHMed framework, as proposed by Devine et al. (2019), is presented as a knowledge-based system composed of three (3) main layers: *Data, Logic*, and *Presentation Layers*, and their associated components. As shown in Figure 2, the framework encompasses data layer, logic layer and application layer. The data layer handles capturing, and storage of the knowledge obtained from the domain experts *via* a data acquisition sub-component and knowledge base. The middle layer—logical layer—embodies the logic that performs reasoning capabilities such as inference and deduction, and extractions of data/information/knowledge based on queries (requests) received *via* the information extraction component. The information extraction component's function is implemented through two sub-components, thus a semantic reasoner and machine learning extractor. The third layer, the presentation layer, presents a means *via* a graphical interface for users to run or pass queries in natural language syntax against the knowledge base to retrieve relevant information on ATHMed. The rationale for the segmentation of the KB framework, is to achieve conformance with current KBS design and, also enable modularity and precision in delivery of a reliable system, serviceable in an integrated, adaptive and platform independent setting, while harnessing the hybrid capabilities (machine learning and ontology specifications) discussed (Devine et al., 2019). This approach conforms to the current software design approaches that seek to adapt an object-oriented focus and is appropriate for reliability, reusability, maintainability and sustainability of semantically focused software design.

### Data Pre-processing

**Figure 3:**
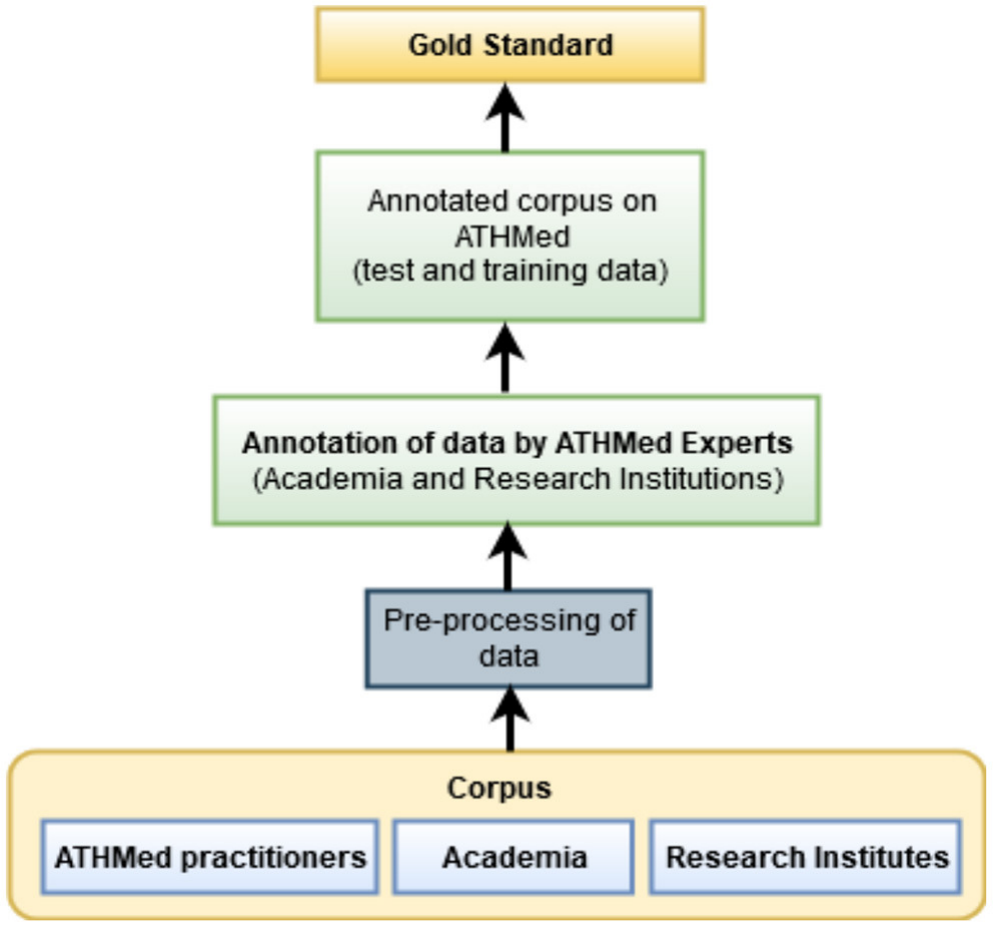
Data sources, preprocessing, and annotation process for ATHMed.

As earlier mentioned in section Conceptualizing the Implementation of ATHMed, the data, captured at data layer stage required for the construction of the KB is to be obtained from the stakeholders which is in agreement with principles for deriving domain knowledge (Studer et al., 1998; Guarino et al., 2009). Since ontologies are focused on building consensus toward a domain knowledge (Studer et al., 1998; Guarino et al., 2009), it is necessary that stakeholders of that domain take critical interest and participate in the building or construction of the knowledge being engineered. As such constant engagement with stakeholders (who are the knowledge bearers) in the process from the information elicitation stage to the building of the final ontology is required. In addition, literature in the domain (which provide explicit knowledge) is also relevant (Boadu and Asase, 2017; Appiah et al., 2019). The initial data (stakeholder knowledge) and relevant information obtained from literature shall be used to build a corpus to train the machine learning algorithm, in hope of obtaining a robust model. Figure 3 shows the data sources for the intended ATHMed domain which include domain experts from academia, research institutions and knowledge from practitioners would be used to build the corpus. This will require preprocessing of the data (unstructured) obtained from the stakeholders for easy annotation.

**Figure 4:**
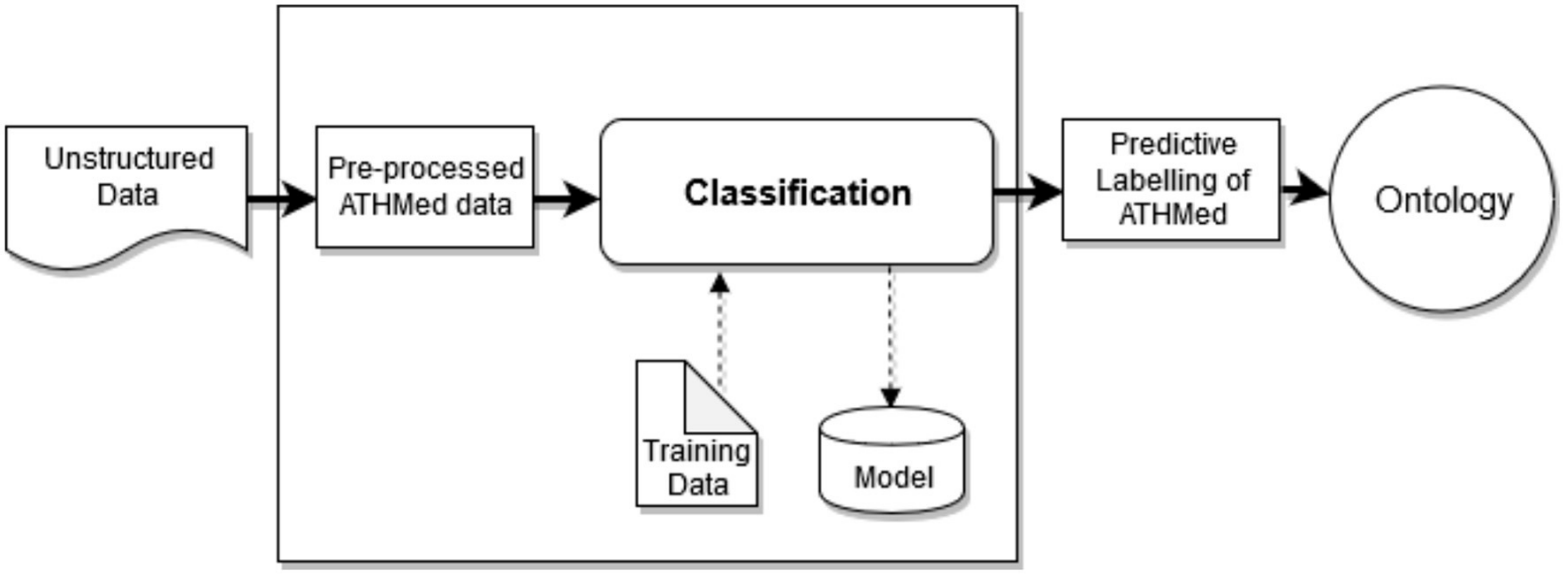
ATHMed classification process.

Preprocessing involves cleaning and conversion of unstructured data into some structured form, reprocessed and then extraction of features where required. This processing is based on the elements relevant for ingredient selection and processing, mode of preparation and administration methods. Through expert domain (academia, research institutions and registered herbal clinics) annotation, a predefine annotation scheme for the ATHMed data will be performed. This will enable the attainment of a good agreement score on what element goes where, to obtain a correctly annotated data for training the machine learning classifier as indicated in Figure 2. The classification process is expected to lead to each data value (or key words) being tagged to allow efficient association of terms and concepts, *via* part-of-speech-tagging (POST) through lemmatization. The processed data, appropriately labeled/tagged will then be stored into the knowledge base considering possible association of data elements, corresponding with the ontology design adopted. By this a reliable level of accuracy in the anticipated prediction and association of data to label is provided as seen in Figure 4. This would include feature extraction and morphological analysis through POST based on some standard medicine preparation template.

Considering the diverse nature of plant/herb types and ingredients, preparatory methods, and administration procedures and the required feature extraction through natural language processing techniques (POST), we suggest an n-gram approach be used. This implies features may be in the form of a unigram, bigram, etc. to aid in storage and retrieval of multiword based expressions and search. The choice of machine learning algorithm to be used in implementing the classifier would require testing several algorithms possibly *via* a machine learning software (e.g., WEKA). Literature indicates that different algorithms have been applied in THMed (Arji et al., 2019) and medical domain (Shang et al., 2017) with varying rates of success. Essential to the machine learning techniques is the indexing approach selected to effluence the storage and retrieval of knowledge (data) into the KB, therefore aligned to the ontology-based KB design proposed.

### ATHMed Ontology Model

The ontology, the ATHMed Ontology model, is to capture all the knowledge into a structured, well-defined form. The ontology approach (computational ontologies) is being adopted to enable the knowledge base possess semantic, extensible and reusable capabilities. The ontology considers the appropriate categorization of classes, anticipated instances, interrelationships and relevant axioms. The ontology approach has become popular in knowledge engineering and representation, yet it is novel in its application in various domains and its potential is still being explored. The ontology requires to be subjected to rigorous evaluation to verify functional specifications thereby ensuring consistency, reliability, accuracy, and extensibility.

To obtain accurate representation, undertake maintenance and efficient dissemination of semantic data (knowledge), the application of computational ontology strategies is proposed and therefore relevant for storage of ATHMed knowledge. In conformance with the DSR method, an additional round of verification on the ontology is required with the aid of domain experts. Hence, a further validation, involving verification and evaluation tests for reliability (Preece, 1994) to ascertain the performance capacity of the knowledge-based system is required. The objective is to measure the ontology's quality to guarantee adequate coverage of the knowledge appropriate to the ATHMed domain.

To derive the ontology, notable metadata representation tools, from authoring to languages for design and writing machine-readable and machine-understandable notation must be employed. This would require the use of Protégé, XML and extensible markup language (XML) Schema, resource definition framework (RDF) and Web Ontology Language (OWL-DL). This is to define appropriate metadata descriptions, structure and storage, and web resources for describing knowledge interrelation. In addition, these tools facilitate the definition of the ontology structure and semantics. Wollersheim and Rahayu (2005) suggest that as information (i.e., knowledge) becomes “explicitly connected”, it evolves consequently becoming “semantically richer”. Toward, the retrieval of data (knowledge) in the ontology-based KB, the value of adopting the ontology strategy is based on the possibility of obtaining reliable, contextual and semantically accurate results. This can be associated with the formalized definition of relationships (Wollersheim and Rahayu, 2005) coupled with the rigidity of constraints. This accounts for the approach proposed in the framework to enhance efficient retrieval of data especially during a search operation. The search and retrieval of the ontology-based data is facilitated through the information extractor component.

### Information Extraction

From the framework, the search and retrieval mechanism are designed to be performed *via* a machine learning extractor that runs query requests against the ontology-based knowledge base. Thus, the information extractor is implemented *via* a machine learning-based (ML-based) extraction algorithm and an inference engine. The overall design is to enable the ML extractor work seamlessly on the ontology to produce semantically oriented results, based on the features stored in the ontology, taking critical considerations of class types, their relationships, instances, connotations and semantics. The ML extractor interacts with the inference engine to deduce new knowledge from the knowledge base (KB) to ensure accurate prediction and determination of appropriate treatment, association to ailment, preparation methods, and other relevant inferences. This, is anticipated, would yield relevant search results as the ontology is formulated on formalized, explicit relationships and axioms, and as such suited for knowledge-based systems especially in knowledge retrieval (Wollersheim and Rahayu, 2005). This, therefore, points toward the need to explore information retrieval strategies and natural language processing (text mining) capabilities which are applicable in knowledge-based systems.

Hersh (2015) views information retrieval (IR) as a field of study that deals with the “acquisition, organization and search of knowledge-based information”. This infers that the information (knowledge) is often derived and organized from research (experimental or observational) and as such focuses on knowledge-based information. Hersh (2015) presents IR as a process that retrieves content suitable to the information needs of a person (user) processed by a search engine *via* a query that attempts to match content items through their metadata, executed based on two sub-processes: *indexing* and *retrieval*. The author further argues that these sub-processes constitute the two intellectual processes associated with information retrieval. Indexing here is referred to as a process that facilitates the assigning of metadata to content items (features). Retrieval, on the other hand, involves the submission of queries posed against the knowledge base that leads to retrieving (search, find and return) of content items (knowledge).

The information extraction's machine learning extractor sub-component is designed to interact semantically with the ontology based on the principle of finding and retrieving knowledge (content items) that fit the search description (query) passed by the user based on the metadata (ontology metadata) of the data (items) stored in the knowledge base. Metadata here focuses on Greenberg's (2003) description of metadata being “structured data about an object that supports functions associated with the designated object”. By implication, for data to be efficiently retrieved an appropriate indexing strategy or mechanism must complement it during storage. Therefore, to obtain optimal results during retrieval of data from the knowledge base, consideration on an efficient indexing strategy suited to both ontology-based and machine learning facilitated knowledge base should be the focus. Additionally, the indexing strategy must be able to influence the retrieval of contextually fit and semantically oriented knowledge. The approach suggested in our framework is targeted at achieving this.

Indexing plays a critical role in the information retrieval process. Indexing can be done manually or automatically (Hersh, 2015), with the latter being preferred due to its obvious advantages of being more efficient, less expensive and applicable to larger and diverse data. Manual indexing involves the use of controlled terminology on a document (data) by human indexers based on some protocol. Automated, thus machine-assigned, indexing deals with the assignment of indexes on terms or words in some document (data). The latter approach is considered in our framework. According to Hersh (2015), indexing occurs by breaking out individual (atomic) indexing units (words, terms or concepts) and assigning them weight based on their frequency in the document (data) and in frequency in other documents. As such indexing is to be carried out at the word or sentence level which is compatible with classification levels and POST. It is expected that this approach will help build an appropriate structure for the data stored in the ontology knowledge base facilitating efficient retrieval.

Several indexing approaches have been suggested in literature. Most indexing approaches are perceived to match terms from some document to those of a query (Soe, 2014). Some of these approaches lean toward providing semantic capabilities suitable for searching ontologies. For instance, Soe (2014) proposed a context semantic index structure that performed superior to other approaches in the retrieval of data based on a context ontology. Therefore, the exploration of ontology-based indexing strategies, applicable to word or text indexing, which support semantic indexing that integrates with machine learning-based extraction mechanisms need to be considered. Additionally, as natural language processing capabilities are suggested in the framework, consideration of word or text indexing approaches is relevant. This is expected to affect how terms/word/features are captured and stored in the ontology-driven knowledge base. Further, it will assist in determining which approach the semantic reasoner deduces knowledge and makes accurate inferences.

The rate of success of the information extractor also is measured by what information/knowledge is retrieved. During retrieval, the relevant information searched and found is returned and/or presented to a user after a given query is submitted. In the retrieval process, an exact or partial match of search approaches can be used with each having its own constraints. However, since a careful annotation of data is to be presented for the classifier to learn, coupled with the need for accurate prediction of terms and concepts notable in healthcare delivery, the exact match search approach would be most suitable. This approach is considered to achieve a more precise prediction of terms relating to THMed knowledge while considering semantics and relevant association of concepts. As earlier indicated, the semantic reasoner presented in the framework acts as the inference engine. The inference engine structure is expected to work on the Java-based framework, Apache Jena. Machine learning techniques mainly to improve the efficiency of predictability, deduction accuracy and an extensible deeper learning approach shall be considered. This shall support the making of inferences whiles deducing new knowledge in a contextual form. This requires that the selected machine learning classifier be trained, initially on some rule-based approach before a well-structured corpus for ATHMed is defined and extended with the algorithm.

Subsequently, as depicted in the application layer, interaction with the knowledge base system is suggested to be performed *via* a graphical interface that is facilitated *via* the web or mobile platform. The aim is to achieve a high level of accessibility as these technologies are deemed ubiquitous and have shown the capacity to support use, anywhere and at any time with ease. Both platforms have the ability to support machine learning and ontology-based applications. Preferably, Android and J2EE technologies are proposed for the design and implementation of the mobile and web solutions, respectively. Users will be provided with simple interactive interfaces that communicative satisfactorily for them to pass simple to expressive statements as queries which will be received as natural language expressions. The use of natural language processing tools and techniques can facilitate efficiently such manipulations. This is to facilitate the extraction of relevant knowledge in the form required for adequate interpretation and understanding to the user. To this end, representation of the extracted knowledge must be textual and visual where required. Similar interface shall be presented to provide new knowledge to the knowledge base.

## Conceptualizing the Implementation of ATHMed

**Figure 5:**
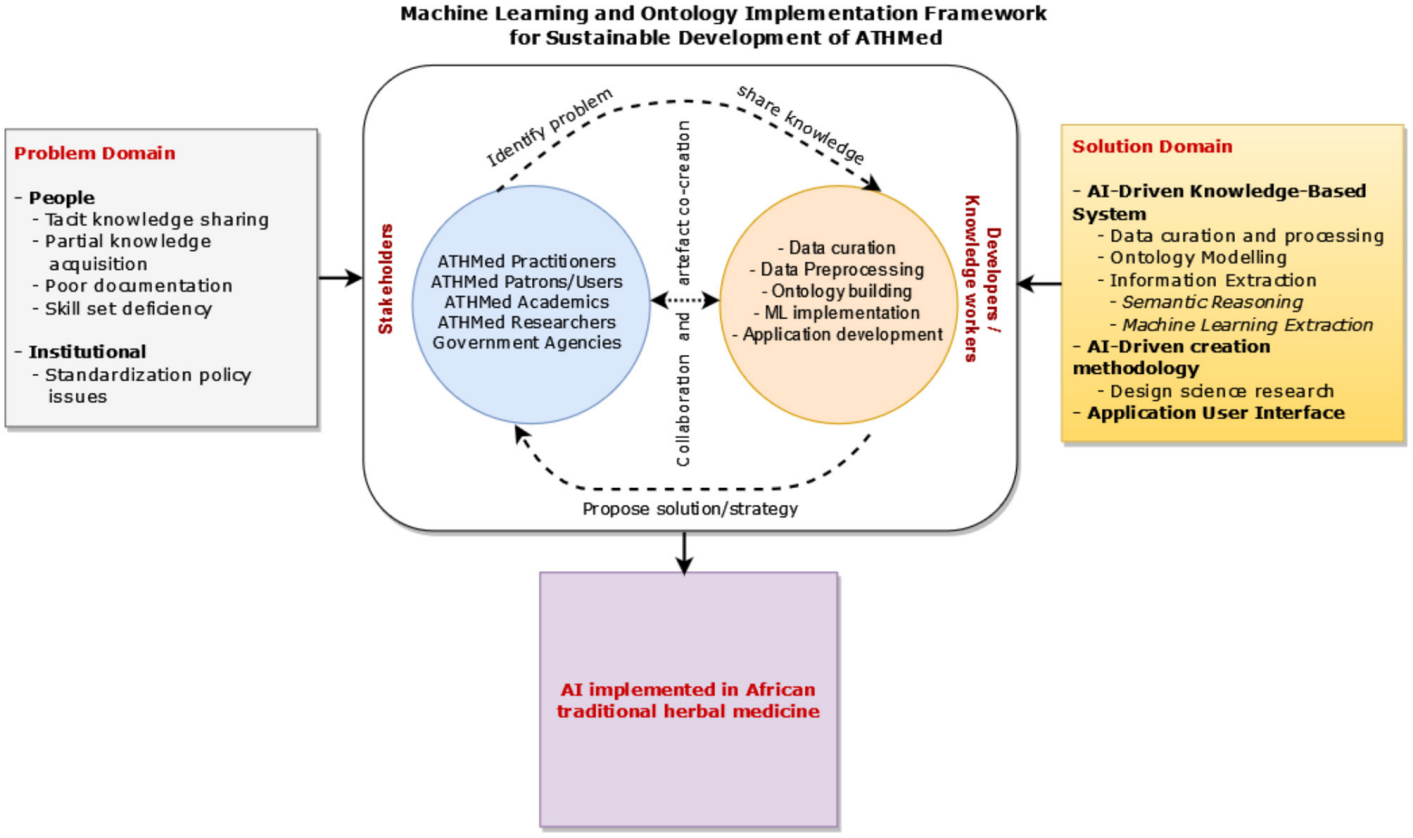
Conceptual framework for machine learning and ontology driven sustainable development of ATHMed.

Based on the core components discussed in the previous sections, a conceptual framework is presented. The framework seeks to provide an approach toward the implementation of a hybrid AI-driven (ML and Ontology) sustainable development of African traditional medicine. Figure 5 presents a combination of problems existing in the ATHMed domain and the proposed sustainable solution. The conceptual framework has three main aspects that are serially aligned: The problem domain, the proposed solution, and the expected artifact. As earlier elucidated, the people in the problem domain are the stakeholders within the ATHMed environment who are practitioners, patrons/users, academics, researchers, and governmental agencies, who motivate the implementation of the KBS in ATHMed. The solution component proposes the KBS implementation strategy that considers the hybrid AI-driven approach in ATHMed. This proposed solution, as suggested in this study, considers a stream of data sources for ATHMed that are pre-processed and annotated to build the ontology model, constituting the knowledge base, suitable for efficient, contextual, semantic knowledge extraction by stakeholders.

The framework envisages an effective and sustainable approach to safeguarding, promoting, and disseminating ATHMed knowledge. The pragmatic approach to identifying, capturing, storing, and retrieval of knowledge on the preparation, application (use), and dissemination of traditional medicines will ensure its continuity, especially for the indigenous use of ATHMed. As earlier discussed, tacit knowledge of ATHMed held exclusively by some traditional healers can be captured and preserved using intelligent computing approaches such as knowledge-bases with AI approaches. Such an approach will enable the use of explicit ATHMed knowledge for formally training health workers in traditional medicine preparation and treatment. It is also geared toward facilitating the discovery of new medicinal properties of medicinal herbs and plants, and helping in advancing research into producing new medicines to serve the healthcare needs of ATHMed patrons. Potentially, this may lead to the conservation and preservation of flora and fauna that could be potential medicinal plants and thereby helping to sustain the local ecology of the communities where these herbs and plants are found. This, in effect, yields a more sustainable approach to healthcare delivery in the African context. Additionally, where the ATHMed knowledge bearer dies, the knowledge still exists in the KBS for future practitioners to learn, harness, and extend the practice of traditional herbal medicines with the needed scientific rigor that ensures safe and standardized preparation and use of ATHMed. This requires consistent collaboration between stakeholders within the domain and AI technology solution providers. This requires consultative engagement involving participatory problem identification, knowledge sharing, and feedback on solutions for ATHMed challenges. Thus, the concept of co-creation of a KBS based on a robust ML and Ontology strategy with stakeholders forms the basis of a sustainable approach to preserving and disseminating knowledge in the ATHMed domain. Consequently, this will help shape learning and training, drug preparation and standardization, and policy formulation within the ATHMed domain.

## Discussion and Limitations

### Discussion

The application of sustainable strategies to promote good healthcare is crucial in maintaining the wellbeing of a person. WHO (2019) reports that 88% of its member states, thus 170 countries worldwide, have acknowledged they use traditional herbal medicine (THMed). It further claims the “uniformly high use” of such healthcare practice further echoes the need to put in strategies, among others, to help ensure safety and monitoring, together with integrating THMed products, practices and practitioners into health systems (WHO, 2019). Accordingly, traditional herbal medicine has proven to be potent and is gaining global attention and use (Frass et al., 2012; WHO, 2019). This could be attributed to its often-natural form, ease of use, availability, and affordability. However, knowledge on these African traditional herbal medicines is gradually being lost if not lost already. The reason is that the knowledge on the preparation and administration of such medication often is tacit, orally communicated and poorly documented as indicated in literature (Yeboah, 2000; Adekannbi et al., 2014; Maluleka and Ngulube, 2018). The possibility of poorly trained ATHMed practitioners, who carry partial knowledge, administering poor medication which has consequentially harmful effects to their patrons is high (Yeboah, 2000). Yet many in the developing countries, especially in Africa, depend on ATHMed for their healthcare (James et al., 2018; WHO, 2019).

To help salvage the situation, deliberate calls and attempts have been made, by academia and research institutions worldwide, to help formalize and train THMed professionals. As custodians, these professionals will not only guide the processes and procedures to the preparation, preservation and administration of THMed, but also ensure the knowledge is not lost (Amoah et al., 2014; Poorna et al., 2014; Boadu and Asase, 2017; WHO, 2019). There are a plethora of reasons to actively pursue documentation, thus preservation and retrieval of ATHMed knowledge (Boadu and Asase, 2017). Poorna et al. (2014) report that countries who have employed meticulous documentation (preservation and retrieval) strategies of their traditional (THMed) practices have yielded immense benefit to them. This reiterates the need for strategies that will help safeguard knowledge on African traditional herbal medicine and thus the proposed ATHMed framework. In essence, the impact of pursuing such THMed knowledge preservation and retrieval leads to key benefits to countries especially in Africa with obvious implications for healthcare benefits.

In an attempt to address the challenges of managing traditional herbal medicine, Atemezing and Pavón (2008) proposed an ontological approach for African Traditional Medicine (ATM) knowledge. Armel et al. (2011) proposed an approach that will enable Clinical Decision Support Systems access to knowledge of traditional doctors and patients to make patient-specific recommendations and provide explanations of their treatment based on deep ontology representation of ATM concepts. Kamsu-Foguem et al. (2013) proposed a framework to improve the formal requirements specification of African TM representation using ontological approaches toward improving the quality of ATM care while ensuring patient safety. Further, Omotosho et al. (2014) proposed a treatment system that used ontology approaches to design and represent Yoruba traditional medicine knowledge for diagnostic and therapeutic purposes. Similarly, Tekemetieu et al. (2016) developed a computer-aided system for ATM that uses ontology to describe knowledge concepts together with a multi-agent system that supports diagnosis and prescription of medicine. Thus, from extant literature, it is relevant to explore the hybrid strategy of ML and ontological approaches to help preserve and share ATHMed knowledge *via* a well-structured, computer-aided, and sustainable basis.

This current study confirms these challenges and finds that the traditional herbal medicine space in Ghana is not well-regulated and guided. The system allows fake and untested herbal medicine that are not monitored in the country. Our precipitants agreed that there is no robust and integrated system for the archival and retrieval of traditional herbal medicine in Ghana. They however blamed these challenges on the mandated regulatory bodies in Ghana. This brings to fore the relevance of the proposed framework in the study. The framework, to the best of our knowledge, is the first to address the challenge by bringing to the fore a *via*ble knowledge-based approach to salvaging potent medicinal knowledge hitherto tacit, facing the potential of being lost in time. The proposed knowledge-based system approach provides a rich-contextual knowledge base on the composition of African Traditional Herbal Medicine (ATHMed) for application and dissemination through a formalized teaching and learning curriculum, while employable for healthcare service delivery. This will form a base for extending research in the area of documenting, preserving, and sharing knowledge on ATHMed and its practices. Machine learning and ontology strategies were presented as a hybrid approach for the framework. This presents a novel approach to tackling issues involving the development of the knowledge-based system that seeks to formally and explicitly preserve and efficiently retrieve ATHMed knowledge in a semantically oriented pattern. The research culminates with an implementation of a framework for ATHMed.

Theoretically, as far as these authors are aware, the framework proposed *via* the hybrid approach is novel in the Ghanaian and largely African context particularly to the application of knowledge-based approaches to safeguarding traditional herbal knowledge. The literature reviewed suggests the integration of machine learning and ontology approaches in the preservation and retrieval of traditional medicine focusing on herbal medicine practices. This article, therefore, suggests useful approaches to consider within the knowledge management domain regarding knowledge-based systems and its application in safeguarding critical knowledge assets. Albeit the framework requires development, implementation and evaluation to ascertain its efficacy and veracity, it provides useful insights for further work. Additionally, the framework is targeted at archiving knowledge relevant for research and training of qualified herbal medicine professional who will ensure continuity and formalization of THMed practices in Higher Learning Institutions. The resulting output from academia will feed the pharmaceutical industry with the requisite knowledge on THMed. Causally, it is hoped that the development of the knowledge base will assist in the conservation of biodiversity, discovery of new bioactive agents and investigation of new herbal drugs for treatment (Boadu and Asase, 2017; Appiah et al., 2019). Although the ATHMed ontology to be developed as part of the knowledge-based system is targeted at suiting the Ghanaian herbal medicinal practices, it can be an excellent starting point to build generic KBs suitable in other similar regions as the modes of preparation of drugs and type of ingredients for herbal preparation and its intricacies may differ and cannot be carried in its entirety to fit the context of other group, regions or countries. The focus is to build a contextually fit ontology which will produce optimal results for the archival (preservation and retrieval) of ATHMed with the Ghanaian herbal medicine as the primary focus.

Practically, our framework and the subsequent development of the KBS is to influence how herbal practitioners undertake drug preparation and to assist in standardizing practices toward the formal training of such healthcare professionals. Additionally, the study intends to implement the KBS that will possess the potential of providing Medical Institutions of Higher Learning the needed repository of well-structured knowledge on ATHMed for research and the formal training of potential herbal doctors and pharmacists. In healthcare delivery, it would help address the concerns of lack of standards often attributed to some herbal products. Furthermore, as the knowledge is to be made assessable to all ATHMed stakeholders, policy and standardization issues (Chikezie and Ojiako, 2015) applicable to ATHMed practice can be made available to all *via* ubiquitous and platform-independent technologies as suggested in the framework.

When a healthcare organization has strong and organized financial management plans, it can provide efficient healthcare to all its patients. Contextualizing Artificial intelligence applications in the health sector ensures prudent financial management. DSR encourages co-creation with end-users. Research has shown that end users are more likely to use a system when they are made to be part of the artifact creation process. Developing an artifact without involving end users retrogresses the acceptance of the artifact and this disrupts prudent financial management. It is hoped that the application of the framework would influence further research into safer and standardized herbal medicines which may be more accessible to the needs of the citizenry and thus not require the use of scarce foreign exchange to purchase medicines that could be locally acquired. This will ensure affordable yet quality healthcare delivery to all, especially ATHMed patrons. There are also financial sustainability implications through potential job creation of new business in the herbal medicine value chain. Thus, livelihoods of indigenes of the countries who adopt and implement the framework, have a potential for improvement especially in the area of healthcare delivery while sustaining valuable biological resources. Poorna et al. (2014) indicated in their study that 6 countries who pursued safeguarding their traditional medicinal knowledge inherently promoted saving and securing patent to these national assets and by extension their cultural heritage. Our framework attempts to provide a sustainable approach to promoting long term use and rediscovery of knowledge helping to answer the call by WHO toward preserving THMed knowledge. In essence, the impact of pursuing such THMed knowledge preservation and retrieval leads to key benefits extending from health to cultural and historical relevance and economic benefits (Van Andel et al., 2015; Boadu and Asase, 2017; Appiah et al., 2019). While we seek to practically implement (develop) the framework in Ghana, we encourage other researchers in the domain to also implement or develop the proposed framework with proper acknowledgment.

### Limitations and Future Direction

There are potential limitations that may hinder the implementation of the framework that could be related to human and technological factors. Considering the technological factor, the ability to adopt and apply appropriate technology for the preservation and retrieval of ATHMed knowledge may inhibit the possibilities of exploring the benefits of the AI-driven solution. Regarding the human factor, the willingness or otherwise of knowledge bearers (traditional medicine practitioners) to share their knowledge and also the acceptance and use of the stored knowledge for a more formalized training and standardized preparation of traditional medicine would determine the practical application, and in effect, the successful implementation of the framework. As this study is ongoing, we intend to automate this framework by leveraging the Design science research framework. Thus, stakeholders of traditional herbal medicine will be made to participate in the co-creation process.

## Data Availability Statement

The original contributions presented in the study are included in the article/supplementary materials, further inquiries can be directed to the corresponding author/s.

## Author Contributions

EK, SD, and RA conceptualize the idea of the article, contributed to the drafting of the introduction, the background of the study, and reviewed and edited the article. EK and SD developed the framework and wrote the methodology. All authors contributed to the article and approved the submitted version.

## Conflict of Interest

The authors declare that the research was conducted in the absence of any commercial or financial relationships that could be construed as a potential conflict of interest.

## Publisher's Note

All claims expressed in this article are solely those of the authors and do not necessarily represent those of their affiliated organizations, or those of the publisher, the editors and the reviewers. Any product that may be evaluated in this article, or claim that may be made by its manufacturer, is not guaranteed or endorsed by the publisher.
